# The Diversity of the Intestinal Flora Disturbed After Feeding Intolerance Recovery in Preterm Twins

**DOI:** 10.3389/fped.2021.648979

**Published:** 2021-03-10

**Authors:** Ying Li, Chunhong Jia, Xiaojun Lin, Lili Lin, Lizhen Li, Xi Fan, Xiaoxia Huang, Zhanyuan Xu, Huixin Wang, Fan Wu, Guosheng Liu

**Affiliations:** ^1^Department of Neonatology, The First Affiliated Hospital of Jinan University, Guangzhou, China; ^2^Department of Pediatrics, The Third Affiliated Hospital of Guangzhou Medical University, Guangzhou, China; ^3^Key Laboratory for Major Obstetric Disease of Guangdong Province, Guangzhou, China

**Keywords:** premature infants, twins, triplets, feeding intolerance, microbiome

## Abstract

**Background:** Feeding intolerance (FI) is a common condition in premature infants that results in growth retardation and even necrotizing enterocolitis. The gut microbiome is linked to FI occurrence; however, the outcome after FI recovery is unclear.

**Methods:** Fecal samples were collected from 11 pairs of premature twins/triplets for 16S rRNA gene sequencing. Initial fecal samples were collected shortly after admission, and then every other week until 7 weeks or discharge.

**Results:** After FI recovery, there was no significant difference in the β-diversity of the intestinal flora between the FI group and the feeding tolerance (FT) group. By contrast, there was a significant difference in the β-diversity. Proteobacteria was the predominant phylum in the microbiome of the FI group, whereas Firmicutes was the predominant phylum in the microbiome of the FT group. The predominant bacteria with LDA >4 between the two groups at 13–15 days after birth, 19–28 days after birth, and at discharge were different, with the proportions of Bacillus, Clostridium butyricum, and Clostridium being highest in the FT group and Firmicutes, unidentified_Clostridiales, and Proteobacteria being highest in the FI group. Similarly, there were significant differences in the relative abundances of KEGG pathways, such as fatty acid metabolism, DNA repair and recombination proteins, energy metabolism, and amino acid metabolism, between the two groups (*P* < 0.01).

**Conclusions:** There was a significant difference in diversity of the intestinal flora after feeding intolerance recovery. Feeding intolerance may disturb the succession of the intestinal bacterial community.

## Introduction

Feeding intolerance (FI), a common condition in premature infants, is caused by an underdeveloped gastrointestinal tract or a disruption in intestinal function. It leads to the slow progression to enteral feeding and prolongs the length of hospitalization. More importantly, FI can lead to neonatal necrotizing enterocolitis (NEC), which threats the life of preterm infants (1).

The human gut is the natural habitat for a large and dynamic bacterial community, and the importance of resident bacteria on a host's physiology and pathology is gradually being elucidated. Recent studies have reported that the status of the intestinal microbiome is closely associated with several diseases, including NEC, inflammatory bowel disease, obesity, cardiovascular disease, and cancer (2–4).

To date, few studies have examined the composition of the gut microbial community in premature infants with FI. It has been reported that the intestinal microbiome changes drastically in cases of FI. Specifically, the proportion of Proteobacteria increases, while that of Firmicutes decreases (5, 6). However, no causative agent has been identified for FI. Furthermore, the composition of the intestinal microbiome after FI recovery remains unknown. The main reason for this lies in the complexity of the gut microbiome, especially in preterm infants. At the perinatal stage, the composition of the gut microbial community can be altered by various factors such as the delivery mode, antibiotic exposure, gestational age at birth, route of feeding, and gender (7, 8). In addition, the microbial composition varies greatly in preterm infants (9, 10). Therefore, the search for the causative agent is complicated by differences in the gut microbiome across different infants (11).

A previous study has demonstrated that the development of the gut microbiome is similar between twins, even within the complex environment of neonatal intensive care (12). Studying twins may provide unique insights into the differences in the gut microbial community. In this study, premature twins/triplets were enrolled to explore the diversity of the gut microbiome in the event of FI. To eliminate birth canal contamination, we only included cesarean section cases. Our results indicate that the effects of FI on the intestinal flora in premature infants continues long after recovery.

## Materials and Methods

### Subjects and Design

This study was conducted at the Third Affiliated Hospital of Guangzhou Medical University in Guangzhou, China. Fecal samples were collected from 11 pairs of premature twins/triplets for 16S rRNA gene sequencing from January 2020 to June 2020. Among these premature twins/triplets, eight pairs were discordant, that is, one infant exhibited clinical signs of FI, while the other infant did not. Initial fecal samples were collected in the first 2 days, and then every week until 7 weeks after birth or discharge. Good clinical practice (GCP) guidelines and regulations were followed for premature infants. Parents were well-informed about this study.

### Diagnostic Criteria of FI

Premature infants diagnosed with FI presented with a gastric residual volume of more than 50% of the previous feeding volume, abdominal distension, gastric regurgitation and/or emesis. The disappearance of the aforementioned symptoms was defined as FI recovery (1, 13).

### Exclusion and Inclusion Criteria

The inclusion criteria were as follows: (1) premature twins with a gestational age <34 weeks and a birth weight <2,000 g; (2) delivery in our hospital by cesarean section only; (3) premature infants were assigned to the FI group if they were diagnosed with FI and their condition improved to achieve full enteral feeding after treatment; and (4) premature infants were assigned to the FT group if they were not diagnosed with FI and achieved full enteral feeding during hospitalization.

The exclusion criteria were as follows: (1) infants who suffered from systemic inflammatory reaction, Crohn's disease, inflammatory bowel disease, congenital gastrointestinal anomalies, or necrotizing enterocolitis; (2) infants who had severe asphyxia, sepsis, coagulation disorders, or those who underwent abdominal surgery during hospitalization; (3) infants whose mother had chorioamnionitis or premature rupture of membranes, as well as perinatal medication and illness were unknown; (4) infants whose symptoms reappeared after FI recovery; and (5) infants who had not achieved full enteral feeding during hospitalization.

### Fecal Samples Collection

Fecal samples were collected with disposable sterile fecal collection tubes from soiled patient diapers and delivered to the laboratory in an ice box. Fecal samples were collected from the first defecation until 7 weeks after birth or before discharge. All samples were stored at −80°C.

### DNA Extraction, PCR Amplification, Library Preparation, and Sequencing

Total genomic DNA was extracted using the CTAB/SDS method. The concentration and purity of DNA were assessed on 1% agarose gels. 16S rRNA v4 genes were amplified using a specific primer pair (16S v4 515F-806R) with a barcode. All PCR reactions consisted of 15 μL of Phusion High-Fidelity PCR Master Mix (New England Biolabs), 0.2 μM each of forward and reverse primers, and 10 ng of template DNA. The thermal cycling conditions were pre-denaturation at 98°C for 1 min, followed by 30 cycles of denaturation at 98°C for 10 s, annealing at 50°C for 30s, and elongation at 72°C for 5 min. PCR products was purified with the Qiagen Gel Extraction Kit (Qiagen, Germany). Sequencing libraries were generated using the TruSeq® DNA PCR-Free Sample Preparation Kit (Illumina, USA) following the manufacturer's protocol. The library was assessed using the Qubit® 2.0 Fluorometer (Thermo Scientific) and the Agilent Bioanalyzer 2100. The library was sequenced using the Illumina NovaSeq platform and 250 bp paired-end reads were generated according to standard protocols by Novogene Co., Ltd. (Beijing, China).

### Data Analysis

Paired-end reads derived from the original DNA fragments were merged using FLASH, which merged paired-end reads when there were overlaps between reads1 and reads2. Paired-end reads were assigned to each sample according to unique barcodes. Sequences were analyzed using QIIME software (Quantitative Insights Into Microbial Ecology), and in-house Perl scripts were used to analyze α- (within samples) and β- (among samples) diversity. First, reads were filtered by QIIME quality filters. Thereafter, we used pick_de_novo_otus.py to identify the operational taxonomic units (OTUs) by generating an OTU table. Sequences with ≥97% similarity were assigned to the same OTU. We selected a representative sequence for each OTU and used the RDP classifier to annotate taxonomic information for each representative sequence. To compute the α-diversity, we rarified the OTU table and calculated three metrics: Chao1, observed species, and Shannon index. Rarefaction curves were generated based on these three metrics. QIIME calculated both weighted and unweighted unifrac, which are phylogenetic measures of β-diversity. We used unweighted unifrac for principal coordinate analysis (PCoA) and unweighted pair group method with arithmetic mean (UPGMA) clustering. PCoA generated principal coordinates and visualized them from complex, multidimensional data. To mine deeper data of the differences in microbial diversity between the samples, significance tests were conducted using the *t*-test, MetaStat, LEfSe, Anosim, and MRPP.

### Statistical Analysis

All clinical data of the preterm infants were analyzed using SPSS 21.0 software. Continuous variables were reported as medians (interquartile interval, IQR), and categorical data were presented as ratios or percentages. A *t*-test and chi-square test were used to examine the significance of the differences in pairwise comparisons of the samples. Differences with *P* < 0.05 were considered statistically significant.

## Results

### Basic Information of Premature Twins

The 11 pairs of premature twins/triplets, including five pairs of monozygotic twins, three pairs of heterozygous twins, and three pairs of triplets, met the inclusion criteria. Among these premature twins/triplets, eight pairs were discordant, that is, one infant exhibited clinical signs of FI, while the other infant did not. The other three pairs exhibited no clinical signs of FI. Among the FI group the fecal flora from 5A to 9C failed to amplification. In summary, there were 14 cases in the FT group and nine cases in the FI group. All premature infants were fed with preterm formula after birth due to lack of breast milk. Only two pairs received antenatal antibiotics, and none of them received probiotics during hospitalization. Latamoxef was the most commonly prescribed antibiotic, and piperacillin was used occasionally, that is, if clinical conditions supported its use. The antibiotics were rarely used for more than 14 days. The demographic characteristics and the basic information of the twins and triplets is shown in Tables 1, 2, respectively.

**Table 1:** The demographic characteristics between FT group and FI group.

	**FT group** **(*n* = 14)**	**FI group** **(*n* = 9)**	***P-value***
Birth weight, g, median	1.38 ± 0.33	1.20 ± 0.28	0.181^**^
Gestational age, weeks, median	31.05 (28.60–33.10)	30.00 (28.60–31.50)	0.137^***^
Gender, M/F	8/6	5/4	0.094^*^
FGR (*n* %)	2(14.3%)	1(11.1%)	1.000^*^
Antenatal antibiotics (*n* %)	2(14.3%)	2(22.2%)	1.000^*^
Latamoxef, days, median	7.5 (0–27)	8.0 (0.0–11.0)	0.503^***^
Piperacillin, days, median	0(0.0–10.0)	0(0.0–9.0)	0.354^***^
Total parenteral feeding, days, median	0(0.0–0.0)	1(0.0–3.0)	<0.001^***^
Duration of NICU, days, median	34.0 (23.0–48.0)	49.0 (36.0–58.0)	0.020^***^

*** *Rank sum test was used to test the significance of difference in pairwise comparisons of the samples*.

** *T-test was used to test the significance of difference in pairwise comparisons of the samples*.

* *Chi square test was used to test the significance of difference in pairwise comparisons of the samples. The medians was described with interquartile interval (IQR)*.

**Table 2:** Basic information of twins and triplets.

**Twins**	**GA** **(weeks)**	**BW** **(g)**	**MZ/** **DZ**	**Diagnose**	**Antenatal antibiotics**	**Time FI diagnosed (*d*)**	**Time FI recovered (*d*)**	**Sample** **(*n*)**
1A	29.1	980	MZ	FI	None	3	8	3
1B	29.1	1,380	MZ	FT	None		–	3
2A	30	760	MZ	FT	None		–	5
2B	30	1,185	MZ	FI	None	2	8	5
3A	28.6	1,330	DZ	FT	Cefuroxime		–	4
3B	28.6	1,350	DZ	FI	Cefuroxime	3	7	4
4A	30.2	960	DZ	FT	None		–	4
4B	30.2	1,440	DZ	FI	None	2	8	4
5B	30.6	1,600	DZ	FT	None		–	4
5C	30.6	1,655	DZ	FI	None	1	5	4
6A	33.1	1,490	DZ	FT	None		–	2
6B	33.1	1,600	DZ	FT	None		–	2
7A	32.1	1,540	MZ	FT	None		–	3
7B	32.1	1,800	MZ	FT	None		–	3
8A	31.5	1,380	DZ	FI	None	2	6	4
8B	31.5	1,315	DZ	FT	None		–	4
8C	31.5	760	DZ	FI	None	3	7	4
9A	29	1,135	DZ	FT	latamoxef	3	6	4
9B	29	1,045	DZ	FI	latamoxef		–	4
10A	33.1	1,706	MZ	FT	None		–	2
10B	33.1	1,750	MZ	FT	None		–	2
11A	29.3	1,040	MZ	FI	None	4	8	4
11B	29.3	1,000	MZ	FT	None		–	4

*GA, gestational age; BW, birth weight; MZ, monozygotic; DZ, dizygotic; FI, feeding intolerance; FT, feeding tolerance*.

**Figure 1:**
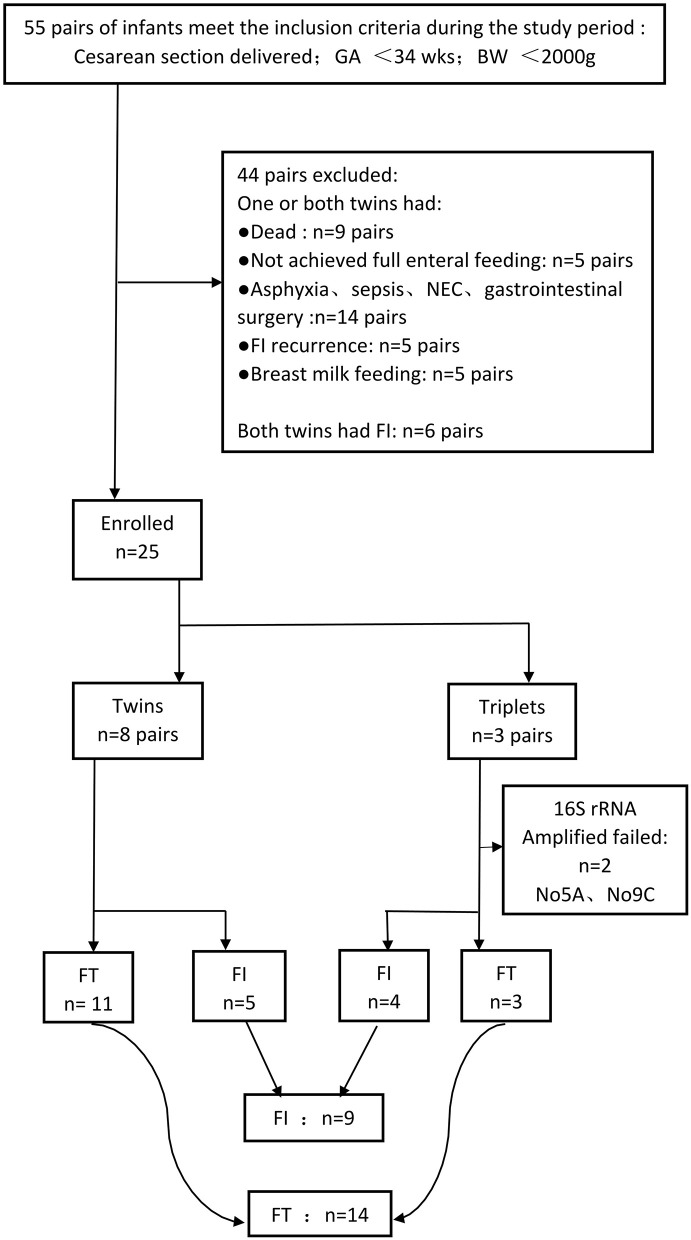
Flowchart of our search.

A total of 131 fecal samples, including 70 in the FT group and 61 in the FI group, were collected. Among them, 33 samples could not be amplified. A total of 82 fecal samples were analyzed, including 46 fecal samples from the FT group and 36 fecal samples from the FI group. The information of the fecal samples is shown in Table 3 and Supplementary Table 1. The flowchart of our experiment is shown in Figure 1.

**Table 3:** The time of sample collection and antibiotics.

	**Samples collection time** **(day)**							**Latamoxef** **(day)**	**Piperacillin** **(day)**	**Diagnosis FI (day)**	**Duration FI (days)**	**TPN (days)**
**1A**	**D3**	**D9**	**D14**	**D21**	**–**	**–**	**D44**	**D1–11**	**–**	**D3**	**5**	**1**
1B	D3	D9	D14	D21	–	–	D44	D1–11 D25–40	–	–	–	–
2A	D2	D9	D15	D21	D27	–	D40	D1–8	D21–28	–	–	–
**2B**	**D2**	**D9**	**D15**	**D21**	**D27**	**–**	**D40**	**D1–8**	**–**	**D2**	**6**	**2**
3A	D3	D7	D15	D21	D28	–	–	D1–9	–	–	–	–
**3B**	**D3**	**D7**	**D15**	**D21**	**D28**	**–**	**–**	**D1–9**	**–**	**D3**	**4**	**1**
4A	D2	D9	D14	D21	D28	–	–	D1–8	–	–	–	–
**4B**	**D2**	**D9**	**D14**	**D21**	**D28**	**–**	**–**	**D1–8**	**–**	**D2**	**6**	**3**
5B	D1	D7	D14	D21	D28	–	–	D1–5	D6–9	–	–	–
**5C**	**D1**	**D7**	**D14**	**D21**	**D28**	**–**	**–**	**D1–9**	**–**	**D1**	**4**	**0**
6A	D1	D7	D14	D21	–	–	–	D1–9	–	–	–	–
6B	D1	D7	D14	D21	–	–	–	D1–9	–	–	–	–
7A	D1	D7	D14	D21	D28	–	–	D1–7	–	–	–	–
7B	D1	D7	D14	D21	D28	–	–	D1–7	–	–	–	–
**8A**	**D1**	**D5**	**D12**	**D19**	**D31**	**D35**	**–**	**D1–4**	**–**	**D2**	**4**	**0**
8B	D1	D5	D12	D19	D31	D35	–	–	D1–10	–	–	–
**8C**	**D1**	**D5**	**D12**	**D19**	**D31**	**D35**	**–**	**–**	**D1–8**	**D3**	**4**	**1**
9A	D2	D7	D13	D21	D28	–	D47	D1–17	–	–	–	–
**9B**	**D2**	**D7**	**D13**	**D21**	**D28**	**–**	**D47**	**D1–10**	**–**	**D3**	**3**	**1**
10A	D1	D7	D14	–	–	–	–	–	D1–9	–	–	–
10B	D1	D7	D14	–	–	–	–	–	D1–9	–	–	–
**11A**	**D3**	**D8**	**D14**	**D21**	**D28**	**–**	**D40**	**D1–8**	**D41–49**	**D4**	**4**	**1**
11B	D3	D8	D14	D21	D28	–	D40	D1–7	D41–48	–	–	–

*1A–11B represented all cases in study. 1A, 2B, 3B, 4B, 5C, 8A, 8C, 9B, and 11A were FI group with bold and the others were FT group. Total parenteral nutrition (TPN)*.

### There Was No Significant Difference in the α-Diversity Between the FT Group and the FI Group After FI Recovery

**Figure 2:**
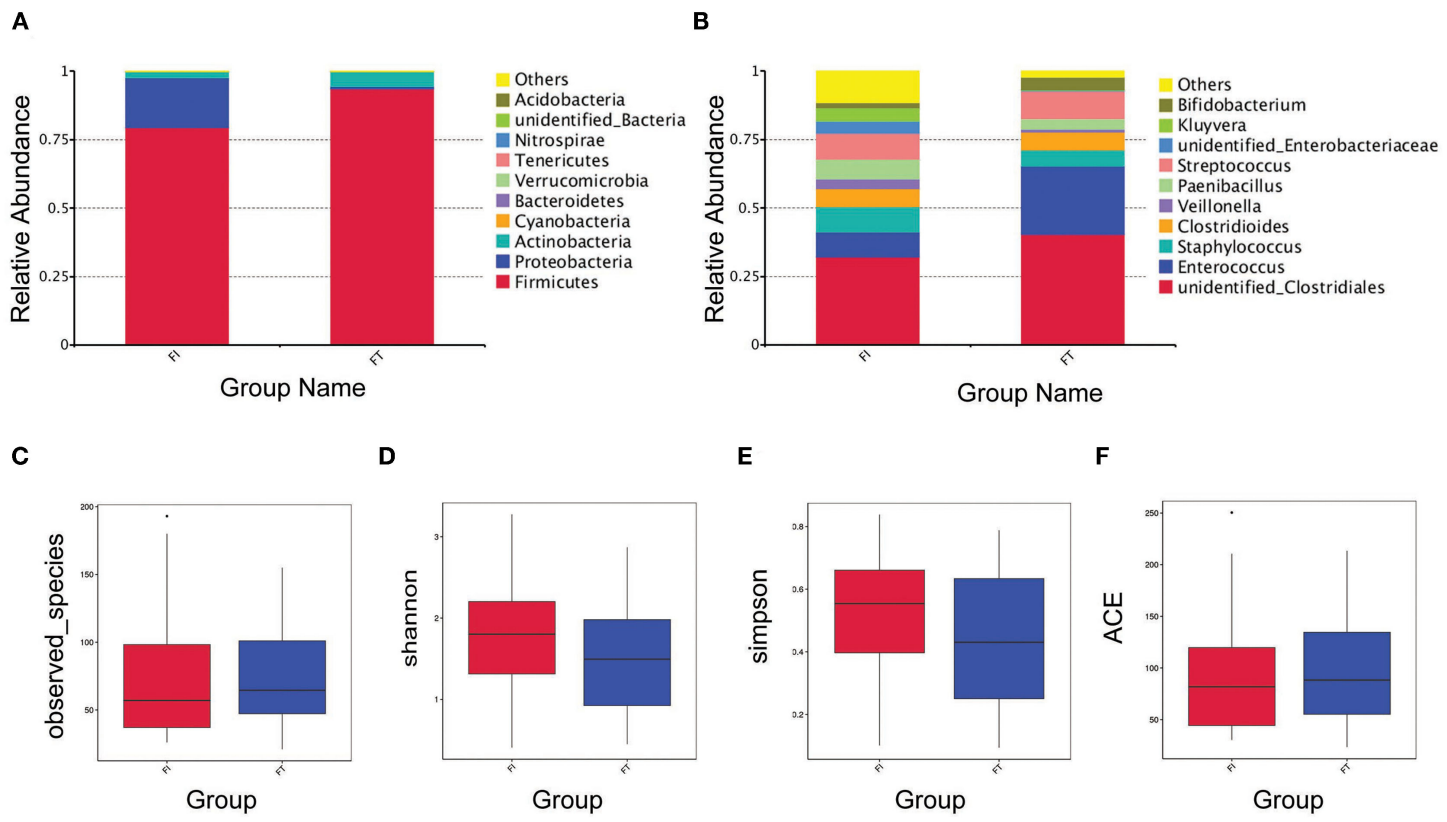
Overview of the preterm infant gut microbiome and the α-Diversity. Relative abundance of bacteria at phylum level **(A)** and at genus level **(B)** between FT and FI groups. α-diversity analysis in the sample groups including observed-species **(C)**, Shannon index **(D)**, Simpson index **(E)** and ACE index **(F)**.

The OTUs, rarefaction curve, and rank abundance curve are shown in Supplementary Figures 1, 2. At the phylum level, the main OTUs in the FT group were Firmicutes (93.63%), Actinobacteria (5.41%), and Proteobacteria (0.81%), whereas those in the FI group were Firmicutes (79.40%), Proteobacteria (18.19%), and Actinobacteria (2.24%), as shown in Figure 2A. At the genus level, the top six OTUs in the FT group were unidentified_ Clostridiales (40.32%), Enterococcus (24.88%), Streptococcus (10.18%), Clostridioides (6.65%), Staphylococcus (5.87%), and Bifidobacterium (4.82%), whereas those in the FI group were unidentified_Clostridiales (32.18%), Streptococcus (9.50%), Staphylococcus (9.44%), Enterococcus (8.90%), Paenibacillus (7.14%), and Kluyvera (4.74%), as shown in Figure 2B. The observed species was similar between two groups as shown in Figure 2C. Additionally, there was no difference in the alpha-diversity presented with Shannon index, Simpson's index and ACE index, respectively, as shown in Figures 2D–F.

### There Was a Significant Difference in the β-Diversity Between the FT Group and the FI Group

**Figure 3:**
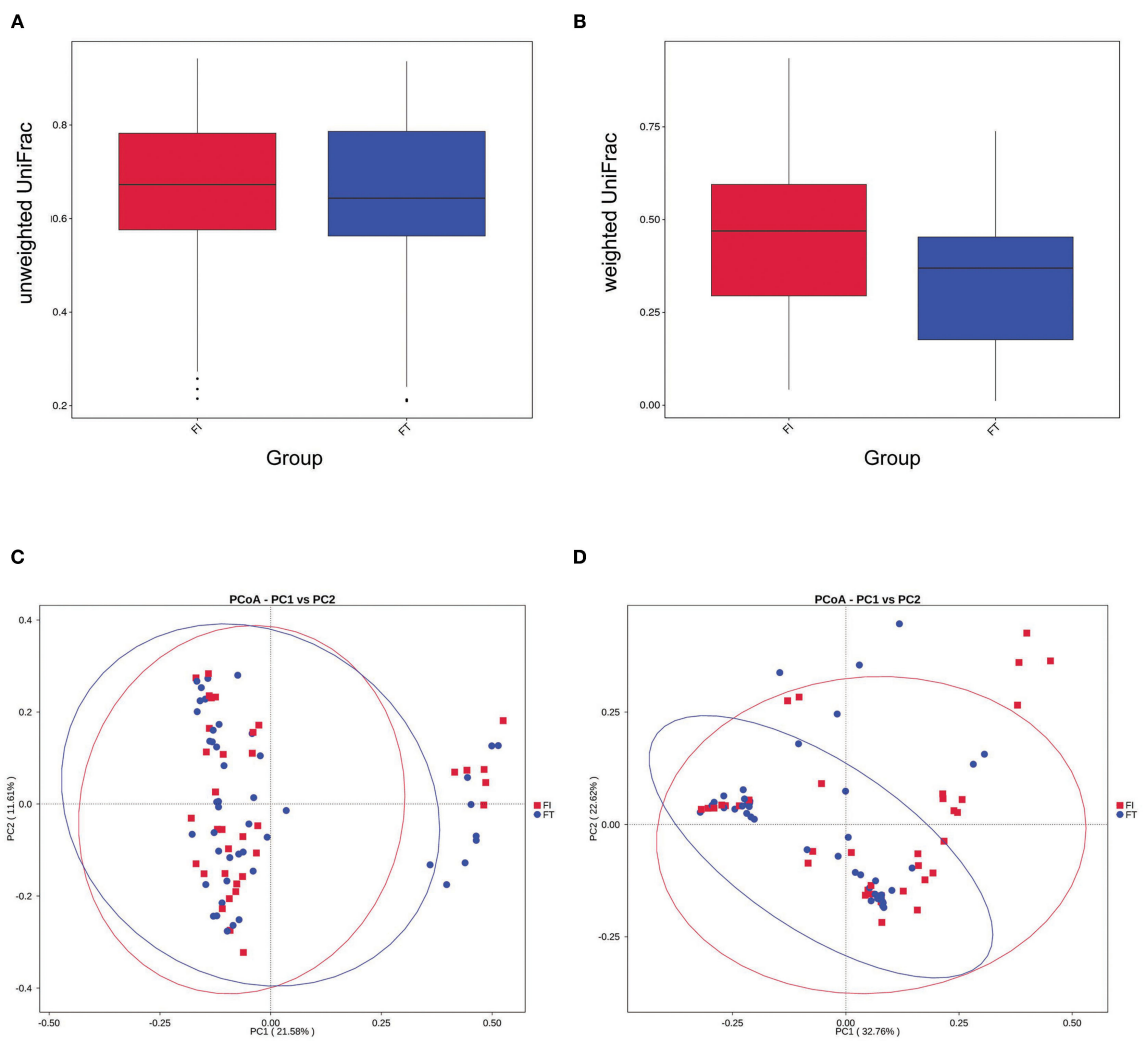
β- diversity analysis. **(A)** Box diagram based on unweighted UniFrac beta diversity *P* = 0.055 (*t*-test) and *P* = 0.040 (two-wilcox). **(B)** Box diagram based on weighted UniFrac beta diversity *P* < 0.001. **(C)** PCoA analysis based on unweighted UniFrac distance. **(D)** PCoA analysis based on weighted UniFrac distance.

**Figure 4:**
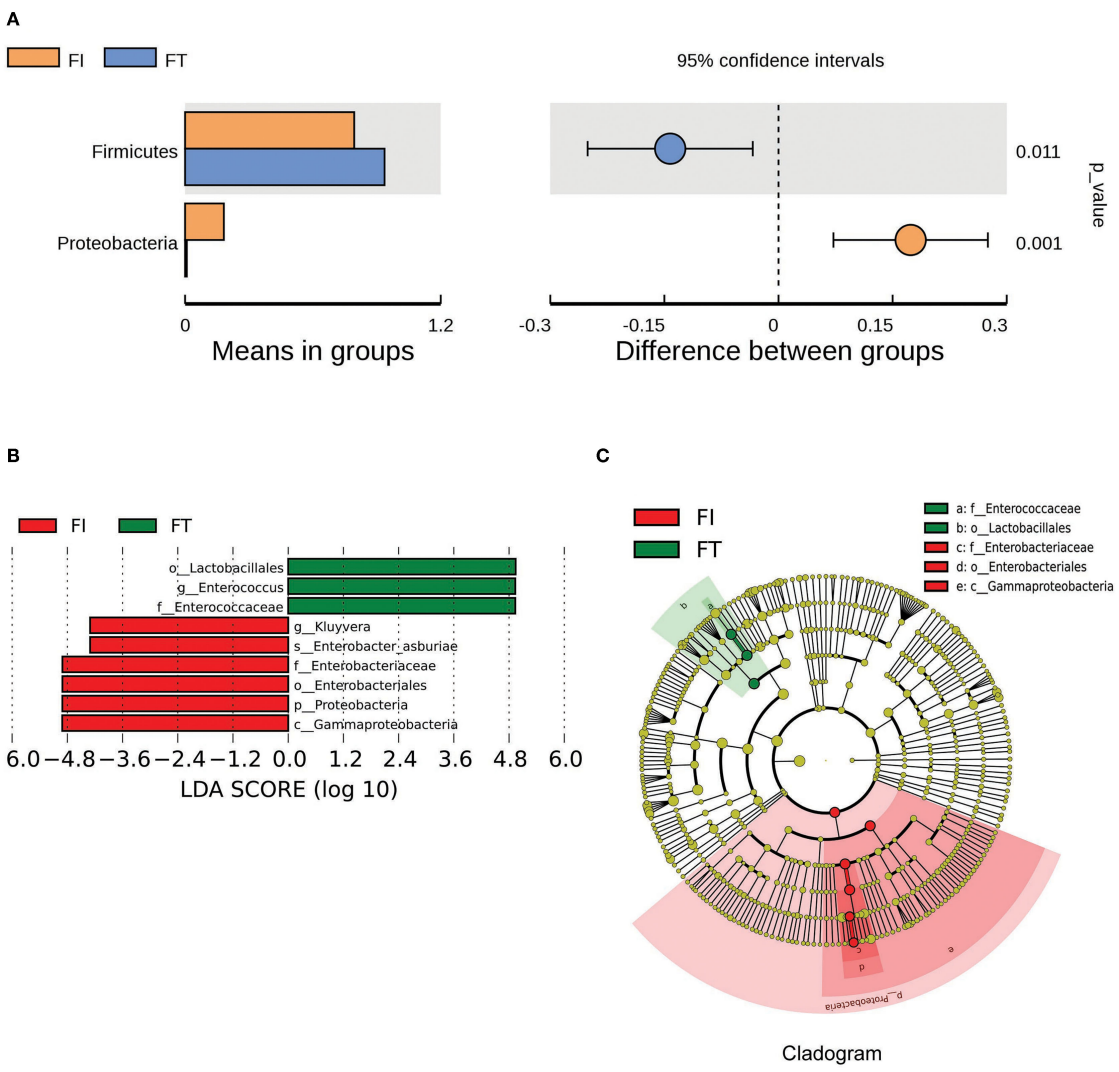
The differential microbes between FI and FT groups. **(A)** Analysis of difference in microbes between two groups using *t*-test. **(B)** LDA value distribution. **(C)** Evolutionary cladistics (phylogenetic distribution).

The box diagram based on unweighted and weighted UniFrac distance showed that there was a statistically significant difference in the β-diversity of intestinal flora between the FT group and the FI group after FI recovery (*P* < 0.05). PCoA analysis showed that similarities in the microbial community in the FT group were higher than those in the FI group (Figure 3). In particular, when the LDA score was set to >4, LEfSe analysis revealed that the predominant bacteria in the FI group were Proteobacteria, whereas those in the FT group were Firmicutes after FI recovery (Figure 4 and Supplementary Figure 3). As mentioned above, not all the twins or triplets were discordant, which means one of the pair suffered FI and the other did not. In 11 pairs of premature twins/triplets, eight pairs of them were discordant. We further analyzed the only eight discordant pairs, as it was shown in the Supplementary Figures 4, 5. There was still significant differences in the β-diversity between two groups based on the eight discordant pairs.

### The Developmental Trajectory of the Intestinal Flora Between the FT Group and the FI Group Was Different

**Figure 5:**
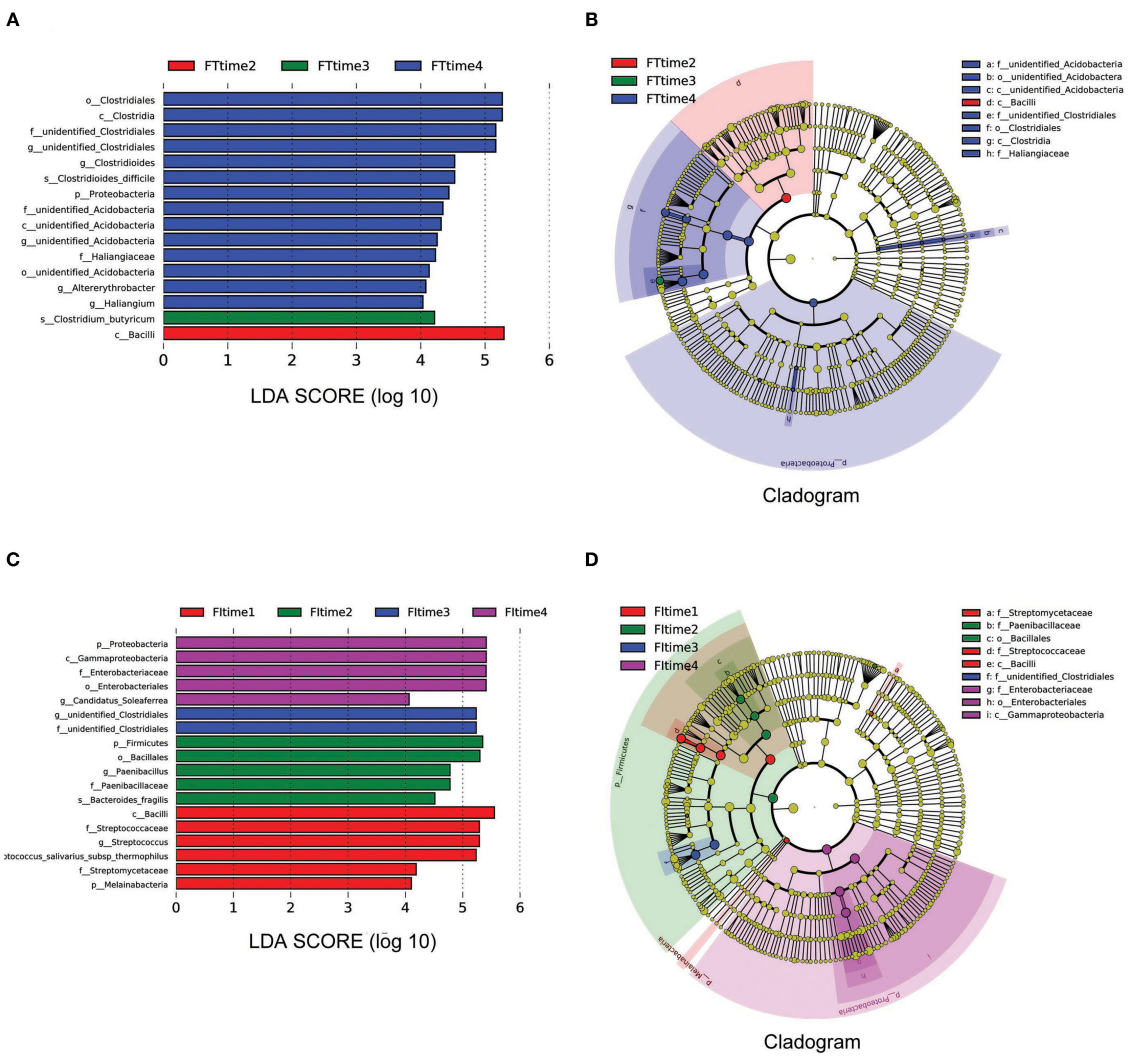
LEfSe analysis based on the different time. LEfSe analysis **(A)** and Cladogram **(B)** in the FT groups over time. LEfSe analysis **(C)** and Cladogram **(D)** in the FT groups over time. Time 1: 7–8 days after birth, Time 2: 13–15 days after birth, Time 3: 19–28 days after birth, Time 4: until the time before discharge.

To examine the developmental trajectory of the fecal microbiota after FI recovery, all samples were analyzed according to the collection time. The samples collected at 7–8 days after birth were grouped as time 1, 13–15 days after birth were grouped as time 2, 19–28 days after birth were grouped as time 3, and the remaining samples were grouped as time 4. LEfSe analysis revealed that the predominant bacteria with linear discriminant analysis (LDA) >4 in the FT group were Bacillus, Clostridium_Butyricum, and Clostridiales at time 2, time 3, and time 4, respectively. However, the predominant bacteria with LDA >4 in the FI group were Firmicutes, unidentified_Clostridiales, and γ-Proteobacteria at time 2, time 3, and time 4, respectively, as shown in Figure 5. Taking into account the differences in body weight and gestational age of subjects, stratification analysis was further conducted. According to weight or gestational age, it was found that there was still a statistically significant differences in the flora between FI and FT groups, as it was shown in the Supplementary Figures 6, 7. Because the samples are limited, the conclusion may be not sufficient.

### Bacterial Functions Between the FT Group and the FI Group Was Different

**Figure 6:**
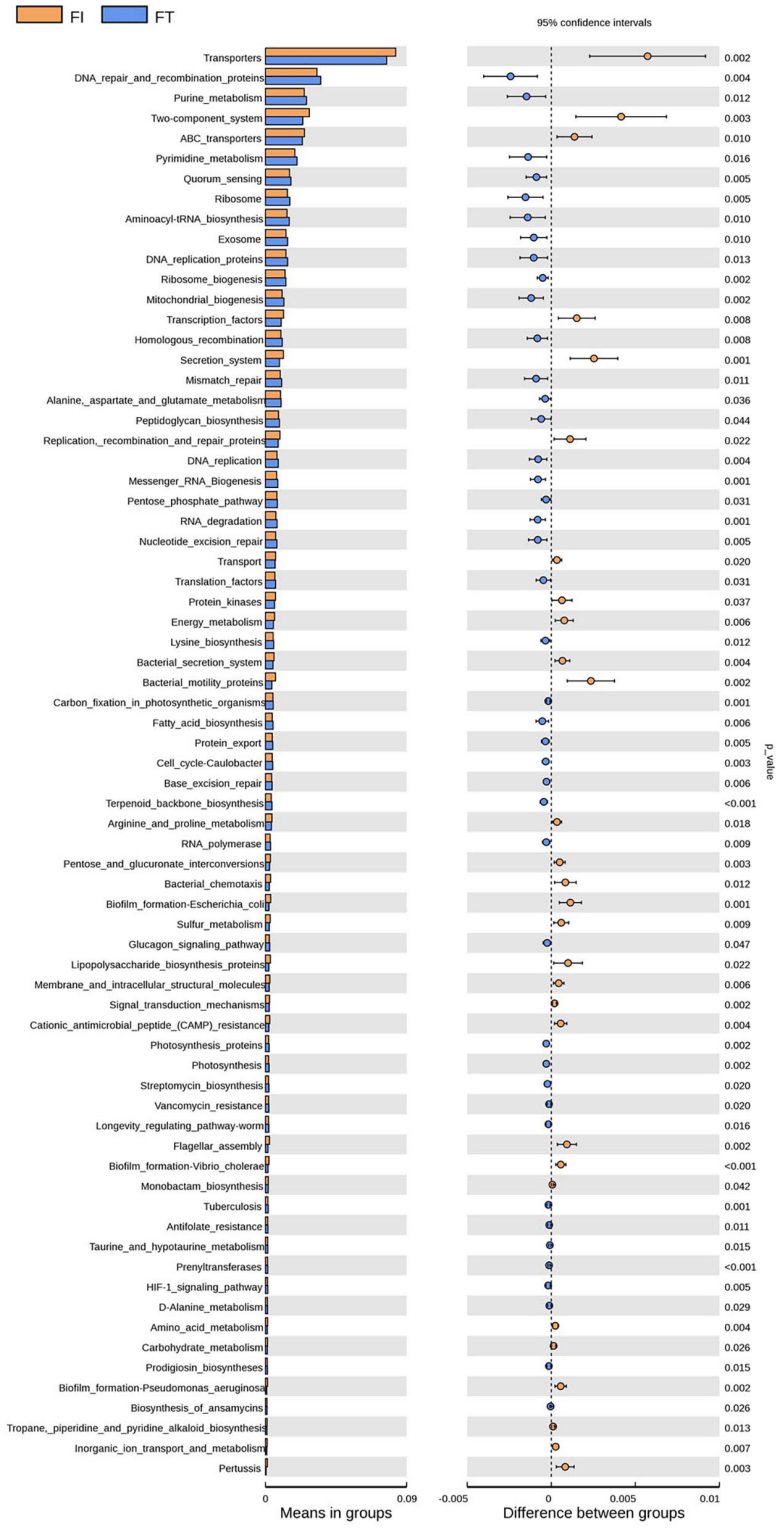
KEGG pathway analysis between FI and FT groups.

Tax 4Fun was used to predict bacterial functions in the fecal samples. After FI recovery, the relative abundances of the KEGG pathways of fatty acid metabolism, DNA repair and recombination proteins, mitochondrial biogenesis, and terpenoid backbone biosynthesis in the FT group were significantly higher than those in the FI group (*P* < 0.01), whereas energy metabolism, amino acid metabolism, bacteria motility proteins, biofilm formation in *Escherichia coli*, cationic antimicrobial peptide resistance, and biofilm formation in *Vibrio cholerae* were significantly higher in the FI group than those in the FT group (*P* < 0.01), as shown in Figure 6.

## Discussion

The initial acquisition and early development of the intestinal microbiome during infancy are important to human health (14). It is now recognized that intestinal microbes are key modulators of disease status. Feeding intolerance (FI) is a common condition in preterm neonates, and it disrupts the homeostasis of the intestinal tract (15). However, the search for its causative agent during early life is complicated due to the mode of birth, antibiotic administration, environment, nutrition, and other individual differences (16). A previous study has demonstrated that microbiome development in preterm twins is similar (12). Thus, in this study, the gut microbiome of premature twins/triplets was examined, where one of the twins exhibited clinical signs of FI, while the other infant did not. To eliminate birth canal contamination, only cesarean section cases were included.

We found that the microbial β-diversity differed significantly between the FT group and the FI group after FI recovery. Enterobacteria from the Proteobacteria phylum were the predominant organisms in the FI group, whereas Enterococcus from the Firmicutes phylum were the predominant organisms in the FT group. As previously reported, there was an increase in the relative abundance of Proteobacteria before and during the diagnosis of NEC and feeding intolerance. *Enterococcus faecalis* plays an immunomodulatory role by decreasing TGF-β and increasing IL-8 levels, and it also regulates the inflammatory response through TLR3, TLR4, TLR9, and TRAF6, which has a protective effect on the body. Our results showed that after FI recovery, the gut environment remained different from preterm infants without FI for at least 7 weeks after birth or discharge, which was also reflected in the different KEEG pathways between the two groups of premature infants. Our results were inconsistent with those of Yuan et al. who reported that Firmicutes was the predominant intestinal flora after FI recovery (6). We speculate the reason for this difference is as follows. Firstly, FI occurred in less than a week in our study, while it occurred at 3–18 d after birth in their study. Secondly, they collected stool samples 1 week after FI recovery, but we collected stool samples from the day of FI recovery to several weeks after recovery. As the development of the gut microbial environment has recently been shown to evolve in a pattern associated with PMA (postmenstrual age, that is, gestational age at birth plus week of life) (17), the different collection times of stool samples, which correspond to different PMAs, may have affected our results.

Our study also found that after FI recovery, the bacterial succession in preterm infants of the FI group was different from that of the FT group in the several weeks after birth. The fecal microbiota of FI infants was dominated by Bacilli at early PMA, followed by Clostridia and γ-Proteobacteria, while that of FT infants was dominated by Bacilli and Clostridia, with the latter class remaining unchanged until 7 weeks after birth before discharge. Grier et al. reported (18) an association between a diagnosis of NEC and microbial reverse transitions from γ-Proteobacteria to Bacilli, Clostridia, and γ-Proteobacteria or a delayed transition from Bacilli to Clostridia. Furthermore, previous studies have revealed that FI and NEC have similarities in clinical manifestations and intestinal flora changes (6, 19). We found that FI may affect the evolution of the gut microbiome in a manner similar to that of NEC. Therefore, enteral feeding of preterm infants that have recovered from FI should be monitored for several weeks after birth.

The accumulated evidence indicates that microbiota development is shaped by host biology and related to the environment. Previous studies have shown that the composition of the intestinal flora of preterm infants was affected by many factors, such as maternal illness and nutritional status during pregnancy (20), delivery mode (21), feeding mode (22, 23), gestational age (24, 25), type and duration of antibiotic use (26, 27), intrauterine or nosocomial infection (28, 29), environment in NCU (30), use of probiotics and so on (31, 32). In our study, we choose those infants that were deliverd by cesarean, no breastmilk feeding, no NEC or sepsis, no probiotics and asphyxia, so we did not analyze those factors mentioned above. As previous studies have shown that the smaller the gestational age, the lower the diversity of intestinal flora, and the later the colonization of anaerobic bacteria such as bifidobacteria and lactic acid bacteria (24, 25). In this study, the range of the gestational age in eight pairs of infants discordant with FI were 28–32 weeks, and the range of the gestational age in three pairs of preterm infants without FI were 32–34 weeks, all of which are extremely premature infants. After preliminary stratified analysis according to gestational age, the main outcome is similar to the original results. Whether gestational age is an important factor that affects the composition of the intestinal flora after FI is cured is not clear, which need a larger sample to conclude. Previous studies have pointed out that the empirical use of antibiotics (including antenatal antibiotics and postnatal antibiotics) can lead to an decrease in Alpha diversity, delayed colonization of anaerobic bacteria, and the impact of antibiotics on the intestinal flora can last several months after the drug was stopped (33, 34). The pre-and post-natal antibiotics used in our study were Piperacillin/cephalosporin. Because there were only two pairs of infants (No 3 and No 5) had antenatal antibiotics, we did not analyze the effect of this factor on the intestinal microflora. In addition, although all preterm infants received either or both of these antibiotics mentioned above after birth, and there were differences in the type and duration of antibiotics usage among different pairs of infants, the type and duration of postnatal antibiotics used between the same pair of twins/triplets are relatively similar, so we did not analyze the intestinal flora of the two groups of preterm infants based on the duration and type of antibiotics under limited numbers.

There were several limitations in this study. Firstly, the sample size was relatively small. However, our study is the first longitudinal analysis of the development of the gut microbiome in premature twins/triplets, where one infant had FI and the other did not. Secondly, this was a single-center study, and it was difficult to eliminate inclusion bias. Thirdly, because most of our initially collected stool samples provided insufficient DNA for sequencing, we only analyzed the stool samples of infants that recovered from FI. Last but not least, we have not been able to compare the intestinal flora of different preterm infants according to the use of antibiotics, but we try to make up for this deficiency by selecting those premature twin/multiple fetuses with similar antibiotic usage. Regardless, the changes in the major bacterial phyla reported in this study are consistent with the current understanding of the effect of those microbes. In conclusion, we found that FI may disturb the diversity of intestinal flora even after FI recovery.

## Data Availability Statement

The authors acknowledge that the data presented in this study must be deposited and made publicly available in an acceptable repository, prior to publication. The data presented in the study are deposited in the (https://www.ncbi.nlm.nih.gov/sra/PRJNA698588, PRJNA698588) repository, accession number (SUB8976175).

## Ethics Statement

The studies involving human participants were reviewed and approved by Institutional Review Board of the Third Affiliated Hospital of Guangzhou Medical University ([2020]009). Written informed consent to participate in this study was provided by the participants' legal guardian/next of kin.

## Author Contributions

FW and GL: conceptualization, project administration, formal analysis, and writing - review & editing. YL and CJ: writing -original draft, writing - review & editing, data curation, formal analysis, and methodology. XL, LLin, XF, and XH: methodology, investigation, validation, and formal analysis. HW and ZX: investigation, validation, and supervision. All authors contributed to the article and approved the submitted version.

## Conflict of Interest

The authors declare that the research was conducted in the absence of any commercial or financial relationships that could be construed as a potential conflict of interest.
