# Supplementary material for: The Diversity of the Intestinal Flora Disturbed After Feeding Intolerance Recovery in Preterm Twins
Source: Front Pediatr. 2021 Mar 10;9:648979. doi: 10.3389/fped.2021.648979 (PMC8006277; doi:10.3389/fped.2021.648979)
Supplement: Supplementary file 1 [file Data_Sheet_1.PDF]

Supplementary material

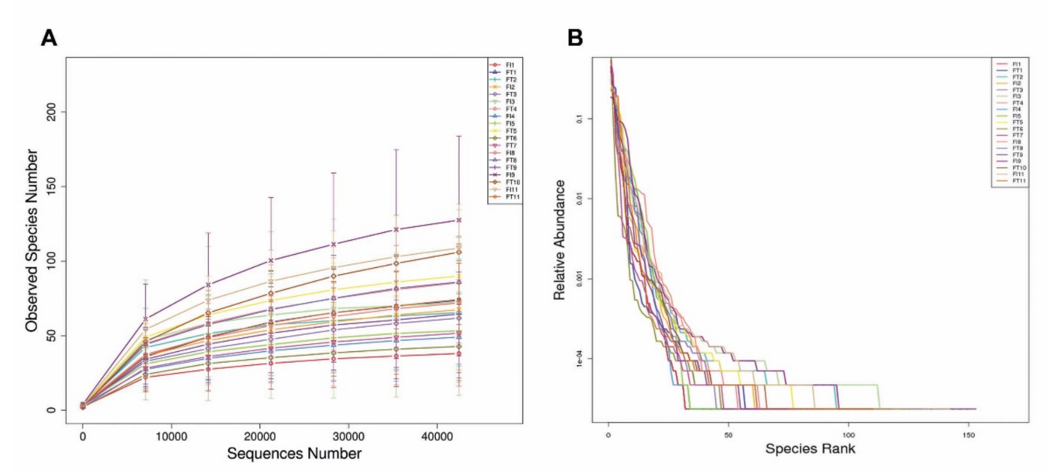

Supplementary Figure 1 The Rarefaction Curve (A) and Rank Abundance Curve (B).

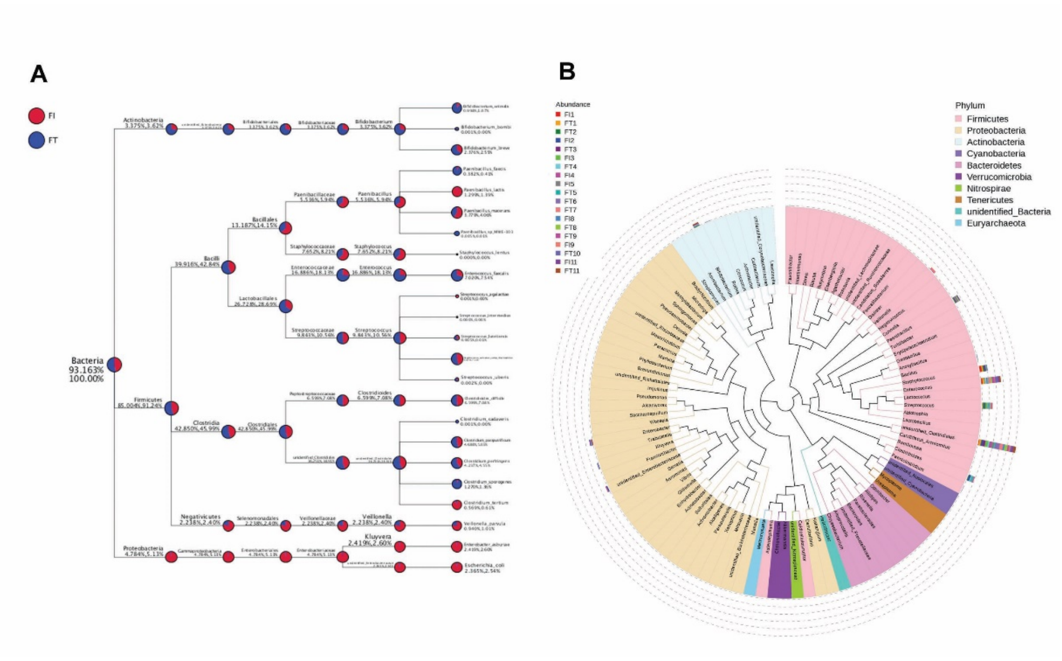

Supplementary Figure 2 The species taxonomic trees (A) and the top 100 species phylogenetic tree at genus level (B).

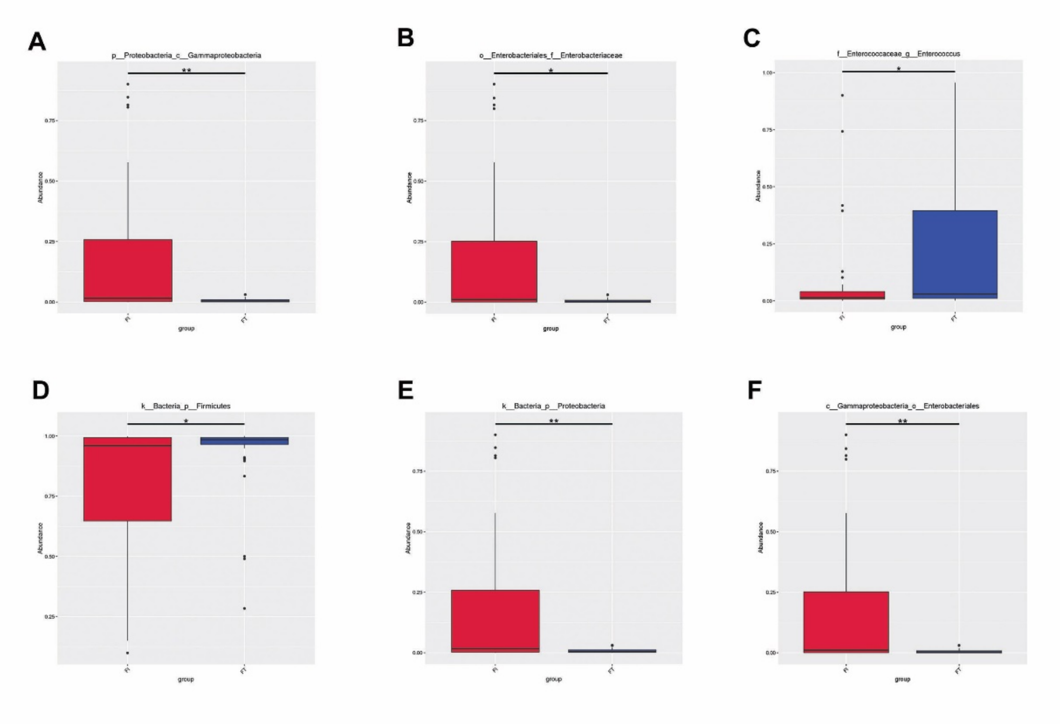

Supplementary Figure 3 Box plot of abundance distribution of different species among groups (A-F).

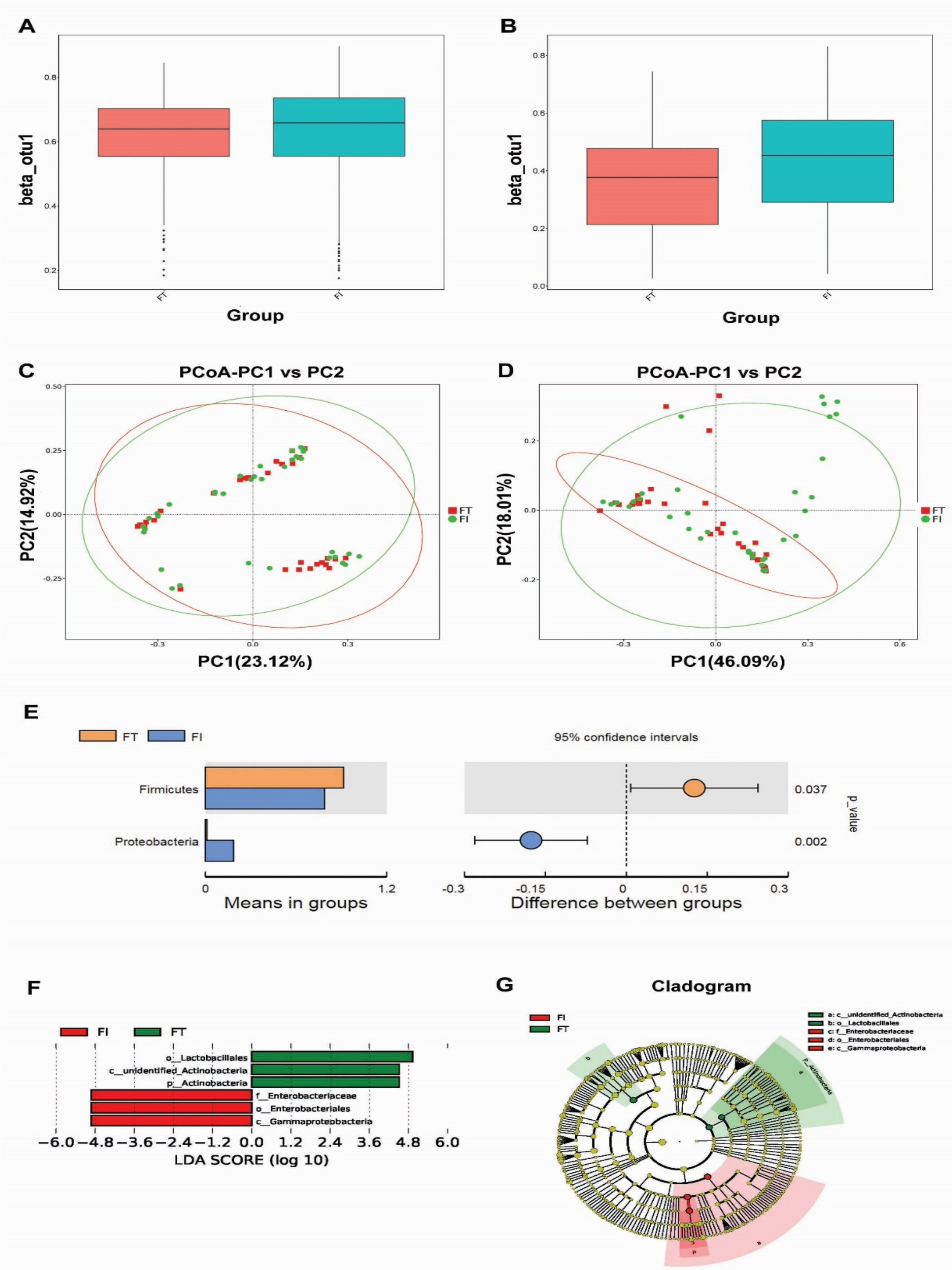

Supplementary Figure 4 β- diversity analysis in 8 discordant twins or triplets (A-G) ：

A：Box plot of Beta diversity（Unweighted Unifrac）；B：Box plot of Beta diversity （weighted Unifrac）；C：PCoA（Unweighted Unifrac）；D：PCoA（weighted Unifrac）；E：Analysis of species differences between groups（T-test）；F：Histogram of LDA value distribution；G：Cladogram

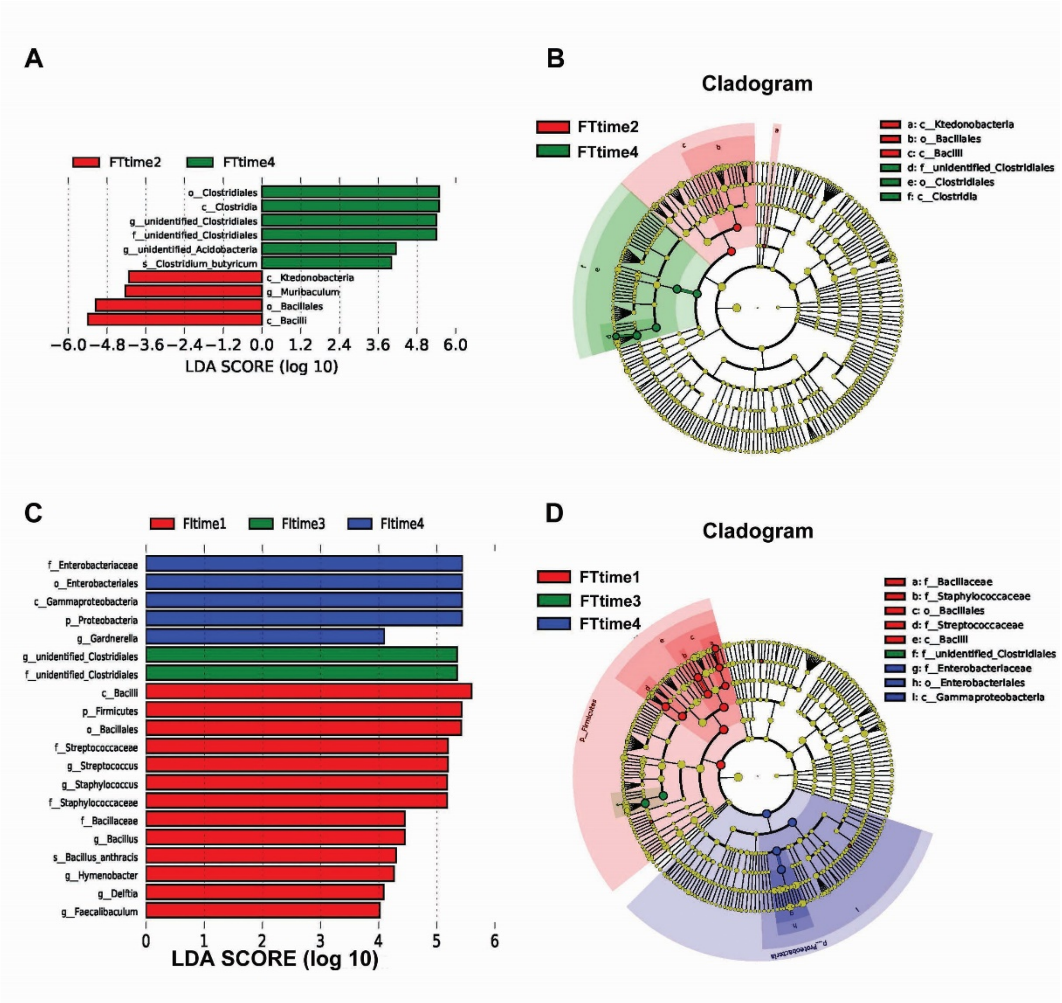

Supplementary Figure 5 LEf Se analysis according collection time between FI and FT groups in 8 discordant twins or triplets (A-D)

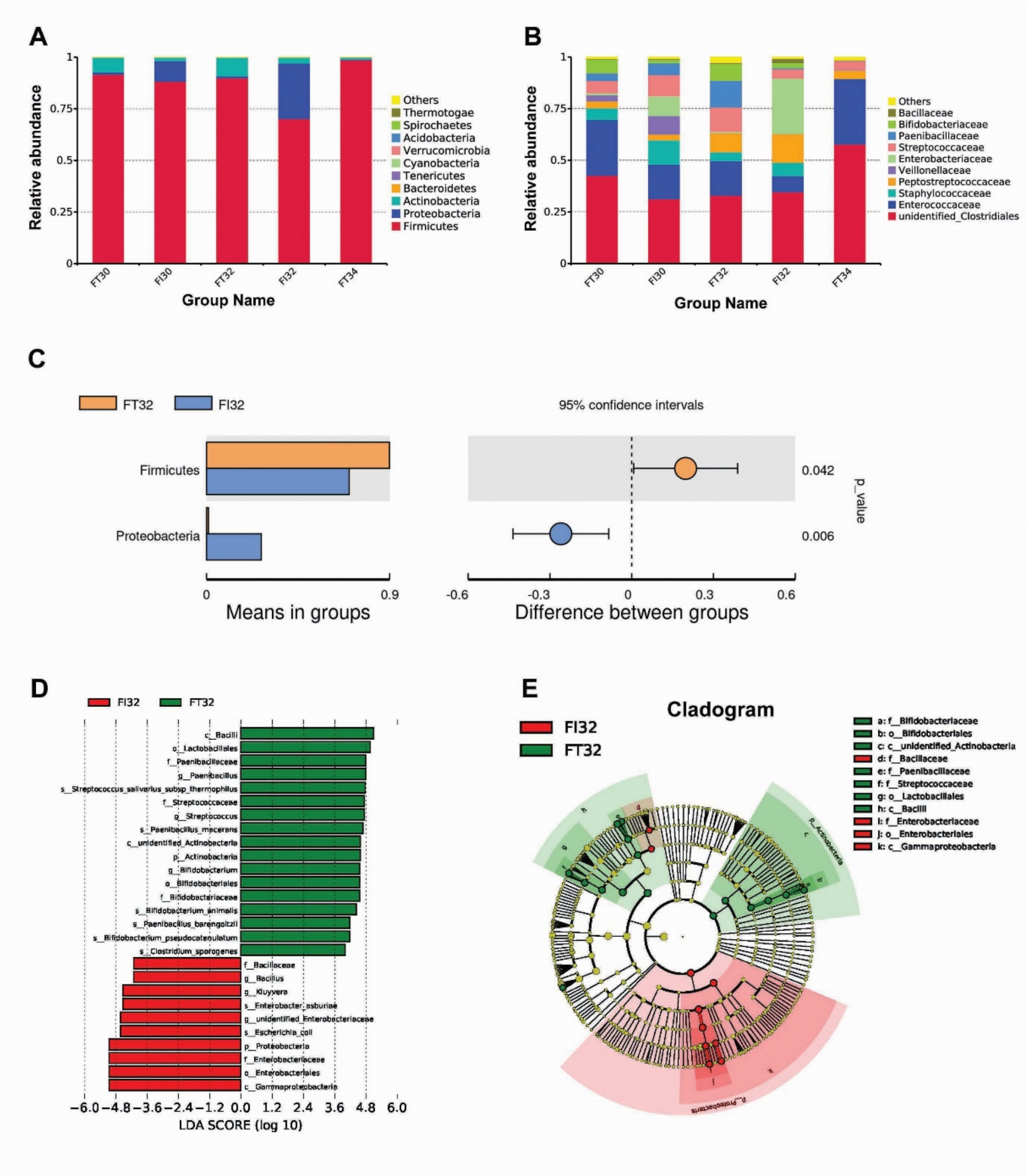

Supplementary Figure 6 The differential microbes between FI and FT groups based on the birth weight (A-F)

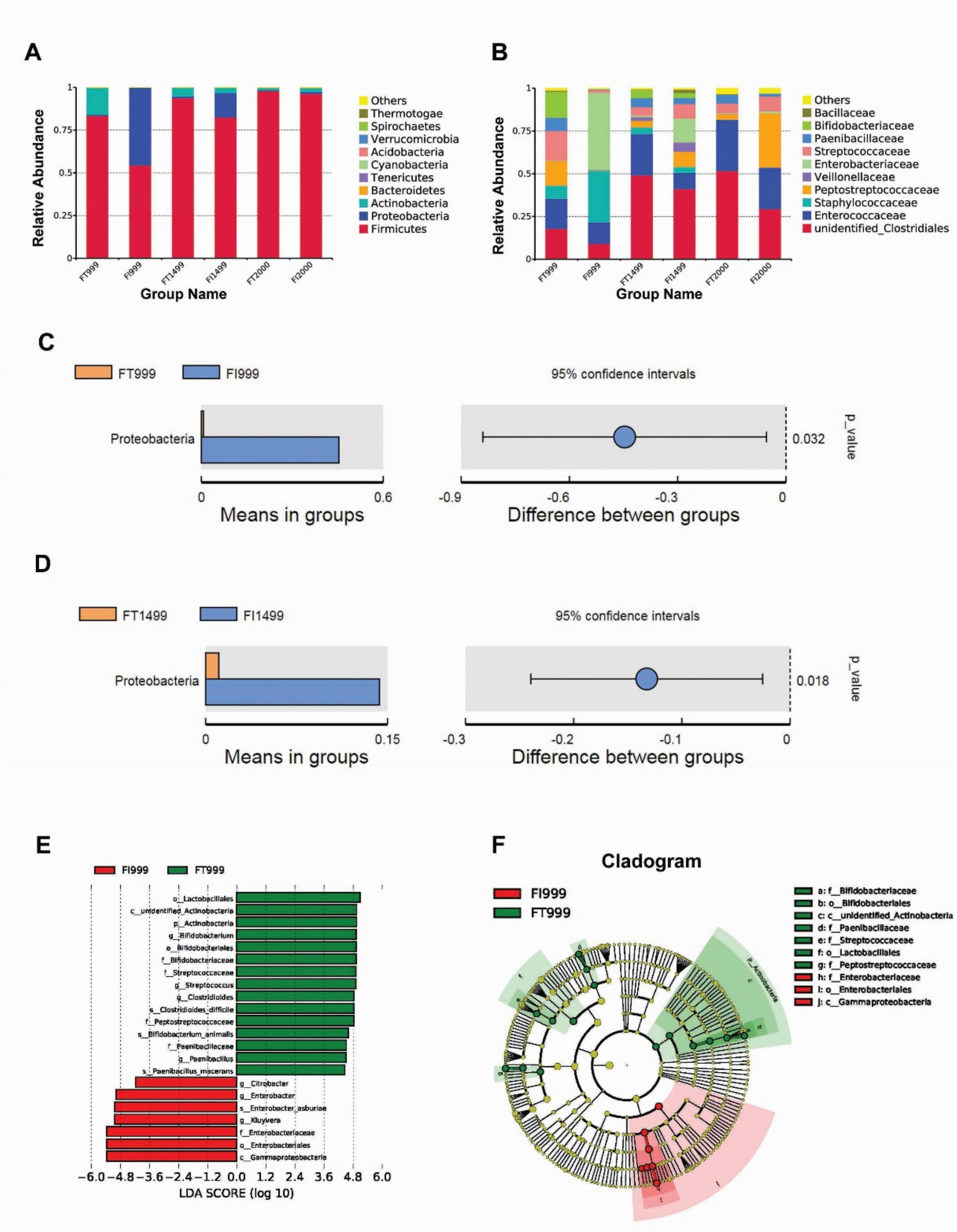

Supplementary Figure 7 The differential microbes between FI and FT groups based on the gestational age (A-F)

Supplementary Table 1 Collection of fecal samples

Time (d)	Fecal samples in FT group		Fecal samples in FI group	
	Total (n= 70)	Amplification failed （n，%）	Total (n=61)	Amplification failed （n，%）
1-2	14	11（78.6%）	11	10（90.9%）
7-8	14	3（21.4%）	11	4（36.4%）
13-15	14	1（7.1%）	11	1（9.1%）
19-21	12	0	11	1（9.1%）
27-28	9	1（11.1%）	10	1（10%）
33-35	2	0	2	0
40-42	3	0	3	0
47-49	2	0	2	0

Time: the time to collect the fecal samples after birth
